# 1965. Promoting COVID-19 Health Equity through Partnership with Community-Based Organizations and Home Vaccination Services

**DOI:** 10.1093/ofid/ofac492.1590

**Published:** 2022-12-15

**Authors:** Justin Daniels, David P Holland, Tanner Forbes, Jane Y Scott

**Affiliations:** Fulton County Board of Health, Gainesville, Florida; Emory University, Atlanta, Georgia; Community Organized Relief Effort, Sandy Springs, Georgia; Emory University School of Medicine, Fulton County Board of Health, Atlanta, Georgia

## Abstract

**Background:**

The COVID-19 pandemic has disproportionately affected racial and ethnic minority groups. The Fulton County Board of Health (FCBOH) sought to promote COVID-19 health equity by partnering with Community Organized Relief Effort (CORE) and Community-Based Organizations (CBOs) to address access barriers and increase vaccine acceptance in Black and Latinx residents. These priority populations were identified using data from the Social Vulnerability Index, the COVID-19 Community Vulnerability Index, and local data regarding COVID-19 positivity and death rates.

**Methods:**

With support from the Centers for Disease Control, FCBOH partnered with CORE to deploy mobile units that provided free at-home vaccination services to Fulton County residents. CORE promoted their services in zip codes identified as having higher proportions of Black and Latinx residents. Additionally, CORE partnered with three CBOs that represented and had pre-existing relationships with our target populations. These CBOs promoted CORE’s at-home services to their constituents. CORE also provided vaccination and health education services at community outreach events hosted by the CBOs as well as at clinics operated by the CBOs.

**Results:**

Between the months of September 2021 and March 2022, our programs were able to provide a total of 897 vaccinations - 510 (57%) at community events, 330 (37%) through at-home services, and 54 (6%) in community-based clinics. Among the 714 vaccinations for which race and ethnicity information were available, 348 (49%) were delivered to Black individuals and 326 (46%) vaccines were administered to Latinx individuals. Uptake of vaccines for Black and Latinx individuals was highest at community events (54% and 88%, respectively), followed by at-home services (35% and 12%, respectively).

**Conclusion:**

At-home vaccination services and partnerships with local CBOs can support a higher proportion of COVID-19 vaccinations administered to populations most at-risk. Notably, community events were the most effective settings for administering vaccines to these populations. Utilizing these methods can help overcome barriers to accessing healthcare and ultimately reduce COVID-19 health disparities in underserved minority communities.

**Disclosures:**

**All Authors**: No reported disclosures.

